# Interdependence of Dosage Form Microstructure and Performance

**DOI:** 10.1007/s11095-017-2137-z

**Published:** 2017-03-15

**Authors:** J. Axel Zeitler, Thomas Rades

**Affiliations:** 1 Department of Chemical Engineering and Biotechnology, University of Cambridge, Philippa Fawcett Drive, Cambridge, CB3 0AS UK; 2 Department of Pharmacy, University of Copenhagen, Universitetsparken 2, 2100 Copenhagen, Denmark

Our understanding of quality in pharmaceutical production has evolved significantly over the past decade. The quality-by-design initiative has proved to be a catalyst for those working in the pharmaceutical science community to reconsider established quality testing protocols and focus research on the effect of processing steps on the quality of dosage forms. With clear progress being made in the implementation of continuous processing into industrial practice there has also been a realisation that pharmaceutical engineering and materials sciences need to be better integrated into existing practice. Traditionally, the pharmaceutical development process, the regulatory process and the testing requirements outlined in the pharmacopeias place a strong emphasis on chemical purity and drug content as well as the safety and efficacy of the medicines. It is this context that has dominated the perception of quality in our community and with which we are all familiar. However, it has become increasingly clear that in order to achieve stable manufacturing processes that result in consistent product quality it is now critical to understand the physical characteristics of drug products in far greater detail than is currently the *status quo*. The microstructure, that is the physical structure at microscopic scale, determines a significant range of critical quality attributes of a dosage form, given their nature as complex multi-particulate mixtures of drug and excipients. Thus there is a continuing need for better fundamental understanding of the interdependence of microstructure and product quality. The lack of sufficiently sensitive measurement techniques and the limited awareness of the role of microstructure for pharmaceutical quality present significant barriers that restrict the production of drug products of the highest quality and within sustainable and economic processes. It is this lacuna that the current themed issue aims to address, through raising awareness, educating and highlighting recent developments that the editors believe have the potential for having a significant impact on the field.

In contrast to the chemical structure of the drug and excipients, the microstructure of the dosage form is defined during the manufacturing process. The same chemical can exhibit a wide range of performance characteristics depending on its physical structure, much like crystalline polymorphs, from a chemical point of view, are identical but will have different solubilities. The role of the microstructure is well illustrated by the example of dissolution testing of immediate release tablets that contain a BCS class I drug. The compendial tests typically require a high fraction of the drug, e.g. 75 or 80%, to be released and dissolved from the dosage form in time periods of 30–45 min. However, closer inspection of the drug release profiles shows that in the vast majority of cases the drug release characteristics vary enormously in the early stages of the test (e.g. at times <15 min) and the variability of the drug concentration only gradually decreases to the ‘acceptable’ threshold close to the defined testing point as specified in the respective compendial method. This variation in release characteristics is directly linked to the disintegration process, which itself is determined by the dosage form microstructure. The review by D. Markl and J.A. Zeitler outlines the fundamental origins of this link and how a lack of meaningful testing protocols has resulted in a lack of appreciation of the connection between physical structure and the quality of the dosage form.

C.C. Sun further develops the argument by highlighting how microstructure not only determines the disintegration of the dosage form but also its mechanical properties that can be achieved by powder compaction. This is of critical importance both in terms of designing the optimal particle characteristics during formulation development and selecting the most appropriate processing route and conditions. The interplay between process parameters, the microstructure and the resulting product performance are critical for defining and evolving the design space during process development and scale up. Even though the formulation is the same, the resulting dosage form can behave very differently. In other words, the microstructure is the key characteristic to understand prior to rational design.

The review by Y. Sun and J. Østergaard complements this discussion by highlighting opportunities that can be realised by replacing traditional UV spectroscopy with modern UV imaging methods for the analysis of the dissolution process. This methodology makes it possible to understand better the interaction between drug and excipients within the formulation and how this can result in different structures (for example in gels, which strongly affect the drug release characteristics of the dosage form). The study by A. Hu et al. highlights this thesis further by providing more in-depth insights into the physicochemical changes that take place within the dosage form during dissolution from a matrix system. Using quantitative magnetic resonance imaging (MRI) experiments and confocal microscopy it is possible to rationalise the structural changes in hydrophilic polymer matrices during drug release. It also sheds light on our understanding of how structural changes affect the mass transport of dissolution liquid into the gel structure and drug out of the dosage form.

An attractive and increasingly popular practice in solid state formulation development is using amorphous drug to increase the bioavailability of poorly soluble drug compounds. But, given that amorphous drug substances are inherently metastable and will eventually convert into a less soluble crystalline form, it is of critical importance to be able to detect crystalline material with sufficient sensitivity and to understand what triggers the crystallisation process. At present, a typical quality control method will attempt to measure the total crystallinity of the entire sample. This is problematic for a number of reasons. Besides the fact that most measurements do not sample the entire volume of a dosage form there is no technique that can reliably detect trace amounts of crystalline material in a bulk sample with sufficient sensitivity. However, even a trace of crystalline material that is spatially confined to a small area of the dosage form can act as a nucleation site from which crystallisation can take place rapidly and throughout the dosage form. The contribution by P.T. Mah et al. provides a fascinating insight into the spatial distribution of crystallinity induced by compression of an amorphous drug substance. The authors were able to demonstrate that, while the presence of excipients did affect the rate of surface crystallisation, it was not able to prevent crystallisation upon tableting. It is important to highlight here that a physical mixture of drug/excipient was used for this study rather than a solid dispersion. The measurements were preformed using a combination of well-established physical surface characterisation techniques such as scanning electron microscopy and attenuated total reflection (ATR) infrared spectroscopy as well as sum frequency generation microscopy (a highly sensitive optical measurement technique that can reliably detect crystallinity of non-centrosymmetric samples).

M. Alhijjaj et al. use the example of an amorphous solid dispersion prepared using a hot melt extrusion process to study the stability of the amorphous drug within the polymer matrix. This combination of formulation and process is one of the preferred industrial platforms for realising amorphous dosage forms. By using advanced thermal analysis routines combined with X-ray microtomography the authors were able to confirm that the crystallisation process is preceded by a phase separation process and the crystal nuclei clearly form from within in the drug domains. The combination of thermal characterisation and spatial resolution of the process provides a unique insight into how the physical structure presets the conditions for the crystallisation process. Following the theme of amorphous stability, K. Punčochová et al. further emphasise how different imaging techniques based on vibrational spectroscopy, as well as MRI, can be used to analyse the drug release process from a solid dispersion formulation. Even if the formulation is stable and the drug molecules are successfully kept in their amorphous state, the solubility advantage can be lost during the drug release process in the patient’s gastro-intestinal tract. This complex process includes mass transport of dissolution liquid into the matrix, swelling, dissolution and supersaturation as well as mass transport out of the drug/polymer matrix. The authors show how it is possible to detect the onset of unwanted crystallisation using the imaging approach during formulation development and how this information can be used to develop a systematic strategy for it.

The next two contributions turn the focus of this theme issue from the formulation and its microstructure at the molecular and domain level to the next larger length scale: the inter-particle scale in granular materials and its effect on tablet structure following compaction. S. van den Ban and D.J. Goodwin describe the development of a high shear wet granulation process to achieve granules with a wide range of densities. This work is a prime example of a quality-by-design driven process development and how this can be realised at production scale. Clearly the density of the granules is of critical importance and largely accords to the microstructure in the subsequent compaction process. Using the tablets produced from these granules as an example, D. Markl et al. show how the bulk porosity of a tablet can be measured non-destructively and without any prior sample preparation using terahertz spectroscopy. The authors argue that porosity is a key critical quality attribute for immediate release tablets, and one that is far better suited to predict disintegration of dissolution characteristics than, for example, tablet hardness.

In the final two articles, attention shifts away from traditional solid dosage forms to emerging drug delivery technologies based on 3D printing. M. Edinger et al. introduce a proof-of-concept study of how Raman spectroscopy and imaging methods can be used in inkjet-printed drug delivery systems to resolve the drug distribution within the dosage form. Finally, with the example of a set of 3D printed dosage forms prepared using the fused deposition modelling technique, D. Markl et al. demonstrate the significant discrepancy in internal structure between the computer aided design models and the actual structures that can be realised using such technology. Whilst there is considerable enthusiasm in the pharmaceutical community to take advantage of the exciting opportunities in 3D printing a meaningful discussion regarding the unique requirements to define dosage form quality is yet to take place and, as demonstrated by this work, microstructure will have to be a central concern.

That new dosage forms and processing techniques require new analytical technologies as meaningful descriptors of quality is a key message expressed by all contributors to this issue. The tools currently described or required by the pharmacopoeiae are no longer fit for purpose as, typically, they do not allow us to quantify the role or effect of the microstructure for the performance of the formulation or dosage form. There needs to be a shared motivation in pharmaceutical development and engineering to develop the tools that the pharmaceutical industry need in order to meet two key challenges of our time: improved drug product quality and a shift to continuous manufacturing. The ability to rationally design, reproduce and reliably detect a well-defined microstructure will be essential to these endeavours, not least to implement meaningful process analytical technologies and control strategies into the manufacturing process.

